# Variables Determining the Postoperative Knee Range of Motion Following Cruciate-substituting Total Knee Replacement: A Prospective Study

**DOI:** 10.7759/cureus.5501

**Published:** 2019-08-27

**Authors:** Nabin K Sahu, Smarajit Patnaik, Saurav Nanda, Mantu Jain

**Affiliations:** 1 Orthopaedics, All India Institute of Medical Sciences, Bhubaneswar, IND; 2 Orthopaedics, Apollo Hospital, Bhubaneswar, IND

**Keywords:** knee replacement, range of motion, cruciate substituting, deformity

## Abstract

Introduction

Range of motion (ROM) is a major desirable outcome after total knee arthroplasty (TKA). The aim of our study is to analyze the variables determining postoperative knee ROM, following cruciate-substituting TKA of arthritic knees.

Methods

One-hundred fourteen patients out of a total of 158 patients were studied on the basis of the inclusion criteria. All patients underwent cruciate-substituting TKA in the period between September 2014 and September 2017. Variables such as age, sex, body mass index (BMI), preoperative ROM, mild-to-moderate knee deformity (in both the sagittal and coronal planes) and knee society score (KSS) were recorded for all the patients. The patients were evaluated at intervals and finally at the end of one-year post-surgery. The final ROM and KSS were noted. Data were analyzed in SPSS system using the Kruskal-Wallis test and Mann-Whitney U test.

Results

Patients with younger age (less than 60 years), more preoperative ROM, and less preoperative fixed flexion deformity (FFD) were found to have better postoperative ROM. The mean preoperative KSS was 42.14 ± 12.02, which improved to 90.86 ± 3.86 postoperatively. Other variables like gender, BMI, and preoperative coronal plane deformity of mild to moderate degree did not have a significant influence on postoperative ROM.

Conclusion

Postoperative expectations of ROM is an important factor for a successful outcome and is also related to patient satisfaction. Variables like younger age group (less than 60 years), better preoperative ROM and lower preoperative FFD are found to have a better postoperative ROM in patients undergoing cruciate-substituting TKA surgery.

## Introduction

Patients with severe arthritis of the knee joint often require total knee arthroplasty (TKA) to reduce pain, improve stability, and restore function. TKA is a highly successful joint reconstruction procedure currently. Surgical outcomes, patient satisfaction, and implant survival have improved steadily since its inception and the procedure has become widely accepted over the last decade [1-5]. The range of motion (ROM) forms an important constituent of most knee scoring systems and thus influences these outcomes after TKA. It is known that 67° of knee flexion is needed for the swing phase of the gait, 83° to climb upstairs, 90° to descend stairs, and 93° to rise from a chair [6]. The minimal flexion of the knee necessary for the usual activities of daily life (ADL) is generally accepted to be 90° [7]. However, some of the ADL require knee flexion in the range of 45° to 105° [8].

It has been shown that the amount of knee flexion significantly influences the total hospital for special surgery knee score, knee society scoring (KSS) system, the stair climbing score, and the walking ability score of patients [9]. While patients hope that their knee range will improve after TKA, this may or may not happen. A large review of TKA using different designs by Tew et al. in 1989 found that 46% of patients could not flex their knees beyond 90° after their surgery [10]. However, with the advancement in prosthetic research, the total knee ROM has increased significantly in recent years [9].

Although arthroplasty has been shown to be successful in the younger population, the majority of the patients, unfortunately, belong to a higher age group, i.e. more than 60 years. In these patients, an uncomplicated arthroplasty would likely last for the rest of their lives. Survival rates for cemented TKA range between 91% and 99% at 10 years and 91% and 96% at 15 years [1-2,5]. Although postoperative ROM following TKA has much importance, there is no definite consensus in the existing literature on the variables that affect it.

The purpose of this study was to analyze the various variables, which could possibly affect postoperative flexion ROM, such as age, sex, body mass index (BMI), preoperative ROM, mild-to-moderate sagittal and coronal deformity, and the KSS.

## Materials and methods

The study was a prospective study, conducted at a tertiary care hospital in eastern India from September 2014 to September 2017. Ethics approval was obtained, as was written informed consent before recruiting patients. Out of a total of 158 patients, 114 patients were included in the study on the basis of the inclusion and exclusion criteria. Patients with osteoarthritis etiology, planned for unilateral cruciate substituting posterior stabilized (PS) design knee implants were included in the study. Patients who were operated for any other prosthesis, such as a rotating platform, high flexion knee, hinged prosthesis, or revision implants were excluded from the study. Similarly, patients with a preoperative bone defect in the knee joint, infected knee joint, and with coronal plane deformity with varus of more than 20° and valgus of more than 15° were omitted from the study. All patients were operated by a single surgeon using the standard medial parapatellar approach. The prosthesis used in our study was cruciate-substituting posterior stabilized design (PFC of Depuy, Johnson and Johnson, USA).

Various preoperative demographic data, such as age, gender, and BMI, were noted. Preoperative ROM and deformity (in both the coronal and sagittal planes) of all patients were calculated by goniometry and divided into subgroups. Preoperative KSS was also recorded. Similar postoperative rehabilitation was carried out for all the patients, both in the hospital and at home. The patients were reviewed at six weeks, three months, six months, and one year. The final postoperative ROM and KSS scores at one year were calculated and compared with preoperative values.

Data collected during the study were entered into the SPSS 16.0 software (IBM Corp., Armonk, NY, US) licensed to our institute for statistical analysis. At the outset, the ROM measurements were tested for normality using the Kolmogorov-Smirnov test. Descriptive statistics like the mean, standard deviation, median, first quartile, and third quartile of the scale variables like age, preoperative ROM, postoperative ROM, and KSS were computed following the procedure described in the SPSS system. In order to study the pattern of distribution of different variables and the visual appreciation of the comparison of variables, diagrams, and graphs have been appropriately used. Postoperative ROM was compared with all the variables such as age, BMI, preoperative ROM, preoperative coronal plane deformity, and postoperative coronal plane deformity using the Kruskal-Wallis test for more than two independent samples. When the Kruskal-Wallis test indicated significant differences among the groups, it was followed by the Mann-Whitney U test for pair-wise comparison of groups.

## Results

The mean age of males was found to be significantly higher than that of the females (p = 0.025). The age distribution revealed that the proportion of cases is higher in the older age group. Out of 114 patients, 26 were male and 88 were females.

The medians postoperative ROM are 115^0^ and 110^0^ for males and females, respectively, and the p-value is 0.992, which is no significant difference between the two. Preoperative ROM was grouped as 1 (< 75^0^), 2 (75^0^ - 90^0^) and 3 (above 90^0^). Their postoperative flexion ROM revealed 100^0^, 105^0^,^ ^and 110^0 ^in the groups, respectively. There is a clear indication of higher flexion ROM in the postoperative period for higher preoperative flexion ROM. The Kruskal Wallis test also confirms a significant difference in the postoperative flexion ROM among the three groups.

Depending upon preoperative coronal plane deformity, all patients were segregated into three groups. Group valgus (0-15 degree valgus), group mild varus (0-10 degree varus), and group moderate varus (10-20 degree varus). Their postoperative flexion ROM was 110^0^, 112.5^0^,^ ^and 100^0^, respectively. The postoperative flexion ROM was not significantly different among the deformity groups (p = 0.072).

Out of 114 patients, 48 patients were having no fixed flexion deformity (group 1), 50 patients were having 0-10^0^ FFD (group 2), and 16 patients were having FFD more than 10^0^ (group 3). The median along with the quartiles of the postoperative flexion ROM were 110^0^, 110^0^, and 100^0^, respectively, for the three groups. This difference was significant (p = 0.003).

Preoperative and postoperative knee society scores have been compared. The mean preoperative KSS was 42.14 ± 12.02 with a median value of 36.5 (Q_1_ - 45 and Q_2_ - 51) and that of postoperative KSS was 90.86 ± 3.86 with median value 90.00 (Q_1_ - 88 and Q_2_ - 90). This shows that KSS has improved significantly postoperatively. All values are shown in Table 1.

**Table 1:** Variables affecting ROM Variables (age, preoperative ROM, preoperative coronal plane deformity, fixed flexion deformity, and KSS score) affecting postoperative ROM in post-TKA and its significance ROM: range of motion; KSS: knee society score; TKA: total knee arthroplasty

Variables	Sub-Groups	N		1st Quartile (Q_1_)	Median	3rd Quartile (Q_3_)		Mean Rank	P-Value
Age	Below 60	44		110	110	120		36.18	0.008
	Above 60	70		100	100	120		24.49	
	Total	114		100	110	120			
Preoperative Range of Motion	Below 75	14		95	100	100		8.71	0.000
	75 – 90	20		100	105	120		23.75	
	Above 90	80		110	110	120		33.86	
	Total	114		100	110	120			
Preoperative Coronal Plane Deformity	Valgus 0-15 degree	20		100	110	120		30.50	0.072
	Varus 0 - 10 degree	56		105	112.5	120		33.07	
	Varus 10-20 degree	38		100	100	110		22.21	
	Total	114		100	110	120			
Fixed Flexion Deformity	No deformity	48		110	110	120		33.19	0.003
	5 to 10	50		100	110	120		30.70	
	Above 10	16		95	100	100		11.12	
	Total	114		100	110	120			
KSS Score	Preoperative				Postoperative				
	Mean		Median		Mean		Median		
	42.14 ± 12.02		36.5 (Q_1 _45 - Q_2_51)		90.86 ± 3.86		90.00 (Q_1 _88 - Q_2 _90).		

## Discussion

In present times, TKA surgery is a standard modality of treatment for painful arthritic disorders among both younger and older individuals because of advanced implant designs, improved surgical techniques, and better patient education. Noble et al. have stated that the ROM after TKA is an important factor in determining the clinical outcome and the satisfaction of the patient [11]. Younger people undergoing TKA have higher expectations, demands, and secondary goals, apart from pain relief, to achieve a “normal-like” joint function with better ROM to suit their desired lifestyles [11]. ROM has been one of the most important criteria for patients to be satisfied with the surgery and is directly related to the patients being physically active [11]. Variables like age, sex, BMI, preoperative ROM, and sagittal and coronal deformities affect postoperative ROM as mentioned in the existing literature.

Lizaur A et al. found a significant decrease in ROM in patients above 65 years of age [12]. Farahini et al. found a significant correlation (p=0.04) between age and postoperative flexion ROM [13]. Anouchi et al. reported no correlation between age and postoperative knee ROM [14]. Reddi et al. and Harvey et al. found an insignificant (p > 0.05) correlation between postoperative ROM and the sex of the patient [15-16]. We found ROM was higher in patients less than 60 years of age as compared to patients more than 60 years of age. 

Lizaur A et al. showed a significant correlation between postoperative flexion ROM and BMI (p < 0.05) [13]. Shoji et al. studied 192 patients who underwent primary TKA and showed that in the group of patients who had flexion ROM of less than 100°, 78% were obese [17]. In our study, we found there was no correlation of gender or BMI with postoperative ROM.

Harvey et al. [16], Farahini et al. [13], and Manke et al. [18] assessed preoperative ROM and found a significant correlation with postoperative ROM. Reddi et al. found no correlation between preoperative coronal plane deformity and postoperative ROM (p>0.1) [15]. Farahini et al. found a significant correlation (p= 0.007) between preoperative coronal plane deformity and postoperative ROM [13]. Harvey et al. found a significant association between preoperative FFD and postoperative ROM (p < 0.0001) [16]. In our study, we found that better preoperative ROM and lower preoperative FFD resulted in better postoperative ROM. There was also no significant correlation between the mild-to-moderate preoperative coronal plane deformity and postoperative ROM in our study.

Farahini et al. found the mean preoperative KSS was 45.2 and the mean postoperative KSS was 93.7, which was twice the preoperative value [13]. In our study, a similar improvement in KSS was observed, from a preoperative value of 42.14 to a postoperative value of 90.86.

In our study of 114 patients, eight patients had early postoperative knee stiffness with ROM less than 60°. All patients improved with aggressive physiotherapy and achieved more than 90° ROM. Two patients had superficial wound infections and improved after superficial wound debridement and secondary suturing. There was no other complication like thromboembolism, deep infection, patellofemoral instability, neurovascular complication, or periprosthetic fracture in our series.

The limitation of our study is that we have not assessed the postoperative implant position and the postoperative correction of deformity for comparison with postoperative ROM. Here, we have included only one specific implant design with the specific surgical technique of cruciate-retaining procedure. However, a further multicentric study on the use of multiple implant designs and surgical techniques is required to provide a definitive conclusion.

## Conclusions

ROM is an important factor for surgical outcomes. The surgeon should have some idea preoperatively to predict the postoperative outcome for dealing with the expectations of the patients. The younger age group of less than 60 years, with better preoperative ROM and lower preoperative FFD, are found to have better post-operative ROM in patients undergoing TKA surgery. Variables like gender, BMI, co-morbidities, and mild-to-moderate preoperative coronal plane deformity do not show any significant correlation when compared with postoperative ROM.
